# Safe handling of hazardous drugs

**DOI:** 10.1177/10781552221135121

**Published:** 2022-11-13

**Authors:** Kardi Kennedy, Kathy Vu, Nadia Coakley, Jennifer Daley-Morris, Leta Forbes, Renee Hartzell, Darrilyn Lessels

**Affiliations:** 1 Cancer Services, 71459 Kingston Health Sciences Centre, Kingston, Ontario, Canada; 2 Safety Initiatives Systemic Treatment Program, Ontario Health, Cancer Care Ontario, Toronto, Ontario, Canada; 3 Teaching Stream, Leslie Dan Faculty of Pharmacy, University of Toronto, Toronto, Ontario, Canada; 4 Leslie Dan Faculty of Pharmacy, University of Toronto, Toronto, Ontario, Canada; 5 Department of Oncology, McMaster University, Hamilton, Ontario, Canada; 6 Ontario Health's Cancer Care Ontario's Program in Evidence-Based Care, McMaster University, Hamilton, Ontario, Canada; 7 Oncology Pharmacy & Systemic Therapy Suite, 25478 Southlake Regional Health Centre, Newmarket, Ontario Canada; 8 Systemic Treatment Program Ontario Health, Cancer Care Ontario, Durham Regional Cancer Center, Oshawa, Ontario, Canada; 9 71459 Kingston Health Sciences Centre, Kingston, Ontario, Canada; 10 Lakeridge Health, Durham Regional Cancer Center, Oshawa, Ontario, Canada

**Keywords:** Hazardous drugs, guideline, hazardous waste, cytotoxic drug administration, personal protective equipment, hazardous drug preparation

## Abstract

**Background:** This evidence-based practice guideline was developed to update and address new issues in the handling of hazardous drugs including being compliant with NAPRA (National Association of Pharmacy Regulatory Authorities) and USP 800 (United States Pharmacopeia) standards, the use of personal protective equipment and treatment in diverse settings including in the home setting. **Methods:** This guideline was developed from an adaptation and endorsement of existing guidelines and from three systematic reviews. Prior to publication, this guideline underwent a series of peer, patient, methodological and external reviews to gather feedback. All comments were addressed and the guideline was amended when required. This guideline applies to and is intended for all health care workers who may come into contact with hazardous drugs at any point in the medication circuit. **Results:** The recommendations represent a reasonable and practical set of procedures that the intended users of this guideline should implement to minimize the opportunity for accidental exposure. These recommendations are not limited to just the point of care, but cover the entire chain of handling of cytotoxics from the time they enter the institution until they leave in the patient or as waste. **Conclusions:** Decreasing the likelihood of accidental exposure to cytotoxic agents within the medication circuit is the main objective of this evidenced-based guideline. The recommendations differ slightly from previous guidelines due to new evidence.

## Introduction

It is well established that antineoplastic agents help treat people with cancer. However, the improvement in patient outcomes must be weighed against the risk of adverse health outcomes for the healthcare workers who handle them. There is no known safe amount of exposure; therefore, healthcare workers must take proper precautions to minimalize contact with hazardous drugs.^1^ The Working Group of the Safe Handling of Hazardous Drugs Guideline Development Group (GDG) developed this evidentiary base to inform recommendations as part of a clinical practice guideline. This guideline is a revision of the previous 2013 OH (CCO) guideline.^2^ The guideline needed to be updated to conform with the NAPRA standards.

## Materials and methods

The objective of this guideline is to update and address new issues in the handling of hazardous drugs. The Program in Evidence-Based Care (PEBC) is an initiative of the Ontario provincial cancer system, Ontario Health (Cancer Care Ontario). The PEBC mandate is to improve the lives of Ontarians affected by cancer through the development, dissemination and evaluation of evidence-based products designed to facilitate clinical, planning and policy decisions about cancer control.

The PEBC supports the work of GDGs in the development of various PEBC products. The GDGs are composed of clinicians, other healthcare providers and decision-makers, methodologists and community representatives from across the province. All work produced by the PEBC is editorially independent from Ontario Health.

The complete methods, search strategies and review processes used for this guideline can be found online at https://www.cancercareontario.ca/en/guidelines-advice/types-of-cancer/2161

The update for this guideline is comprised of a combination of adaptation, endorsement and three systematic reviews. This update ensures that the PEBC guideline is compliant with NAPRA^3^ and USP 800.^4^ The three systematic reviews were in the areas of closed-system transfer devices, pregnancy outcomes in healthcare workers who handle cytotoxic drugs and general health outcomes in health care workers who handle hazardous drugs. The target population and intended users of this guideline are health care workers who may come into contact with hazardous drugs at any point in the medication circuit. It is also intended for Hospital Administrators, Educators and Managers, Occupational Health and Safety Services.

## Recommendations

In the recommendations that follow, the following action verbs are used to help the intended user determine the level of variation one might expect from following that recommendation.

*Legislation/regulation requires* – A recommendation that is supported by law, regulation or standard. All centres and users would be expected to implement this recommendation with little variation.

*Strongly recommend* – A recommended course of action or practice based on evidence in the medical literature and/or a strong consensus of the expert panel. Variation from this course of action or practice should be based on a considered judgement of how the local circumstances may vary from those typically found in practice.

*Recommend* – A course of action or practice which, in the consensus of the expert panel, is sound and worth considering, but whose implementation may vary according to local circumstances.

Hierarchy of Controls

“Controlling exposures to occupational hazards is the fundamental method of protecting workers,” as stated by The Centres for Disease Control and Prevention in the NIOSH (National Institute for Occupational Safety and Health) Engineering Controls Program Portfolio. It describes the Hierarchy of Controls used to implement feasible and effective controls. In descending order, they are Elimination, Substitution, Engineering controls; Administrative controls and the use of Personal Protective Equipment. “Engineering controls are used to remove the hazard or place a barrier between the worker and the hazard.”^5^ In health care, examples of engineering controls include the use of biosafety cabinets and safety-engineered medical devices (SEMDs): particularly, safety-engineered needles help protect the worker from blood-borne pathogen exposures. Administrative controls include policies and procedures and staff education and training.

Although Personal Protective Equipment is the last control between the hazard and the worker, it really is the primary control on which we rely. It is very important that health care workers are educated in the appropriate selection and use of Personal Protective Equipment for protection against exposure to cytotoxic drugs. This usually consists of the use of gloves, gowns and eye protection as appropriate.

### Recommendation 1: general measures

#### Committee responsible for policy and procedures for hazardous drugs

It is strongly recommended that all institutions administering hazardous drugs form such a committee. It is also strongly recommended that this committee include, but not be limited to, representatives from various departments and services such as occupational health and safety, joint health and safety committee, pharmacy, nursing, medical oncology (physician), environmental services, risk management and a patient representative.

This committee would be responsible for clear processes of developing, reviewing and revising policies and procedures related to hazardous drugs. A risk assessment and gap analysis should be routinely conducted to identify gaps and to inform policies and procedures. In addition, this committee is responsible for ensuring that there is a process in place for orientation and ongoing education for the identified target population. This committee is responsible for the implementation and follow-up of the Risk Prevention Management Program related to the use of hazardous drugs.

#### Continuing education and orientation program

It is legislated that initial and ongoing hospital-approved education be provided to all staff involved with hazardous drugs throughout the medication circuit including safe handling and spill or leak management.^6^ It is strongly recommended that all staff have initial and ongoing training related to best practice standards in place at the time.

It is legislated that there is documentation that annual training on safe handling of hazardous drugs has occurred.^6^ This should be documented by the institution's Committee Responsible for Policy and Procedures for Hazardous Drugs.

#### Identification and safety

It is strongly recommended that each institution maintain a list of hazardous drugs that are used in their facility, that is reviewed regularly, when policy is updated, and whenever a new agent or dosage is used.^4^

It is legislated that hazardous drugs and their waste be properly identified with the symbol capital “C” and, under it, the words “CYTOTOXIC/CYTOTOXIQUE” in capital letters.^7,8^ It is legislated that all hazardous waste under the Ministry of Environment, Conservation and Parks regulation (guideline C-4) include bilingual wording and both the words and the symbol appear on a dark grey rectangle.^7,8^ Other countries may have their own systems for labelling and should be adhered to.


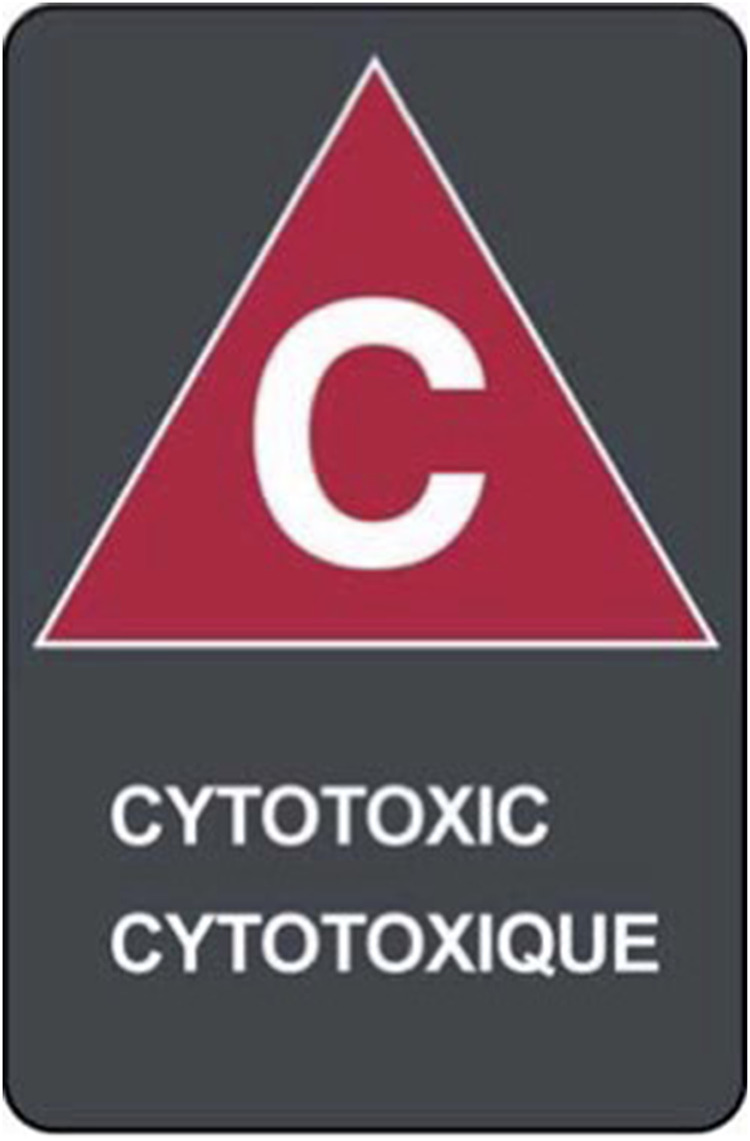


#### Purchasing of drugs

When purchasing hazardous drugs, it is strongly recommended that institutions consider vendors that include safe handling measures such as pre-wiped or protective containers or smaller receptacles to decrease volume of potential spills.

#### Spills kit

It is strongly recommended that a spill-management kit be available in all areas where hazardous drugs are stored, transported, handled and administered.^3^

#### Precautionary reassignment

It is strongly recommended that all staff be fully informed of the potential reproductive hazards of hazardous drugs.^9^ It is strongly recommended that the facility consider alternative duties for staff who are pregnant, breastfeeding or actively trying to conceive.

### Recommendation 2: PPE

It is legislated that a worker works in compliance with the Occupational Health and Safety Act and regulations and use or wear the equipment, protective devices or clothing that the employer requires to be used.^10^

It is legislated that the appropriate PPE for the task (as described in Table 1) be worn throughout the medication circuit.^10^ It is the employer's responsibility to provide the necessary protective equipment and training on how to use the equipment.

**Table 1.** Personal protective equipment to be worn throughout the medication circuit.

**Medication circuit steps**	**Gloves**	**Gown**	**RPD**	**Facial protection**	**Cap**	**Shoe covers**
Unpacking and cleaning	• (2 pairs)	•	• (Only if unpacking hazardous drugs that are not contained in plastic until assessment of the packaging integrity can be made)			
Sterile preparations	• (2 pairs)	•		•	•	• (2 pairs)
Non-sterile preparations: - Counting of solid oral forms	• (1 pair)	•				
Non-sterile preparations: -Preparing creams, ointments, oral solutions and crushing tablets	• (2 pairs)	•			•	• (2 pairs)
Routes of administration (intravenous, subcutaneous, intramuscular, intravesical, intraperitoneal, intrathecal, liquid oral)	• (2 pairs)	•		• (If risk of splashing, e.g., bladder installation or NG, G, or J tube)		
Solid oral administration (tablets)*	• (1 pair)					
Topical administration (creams, ointments)	• (2 pairs)	•		• (If risk of splashing)		
Aerosolized administration (e.g., ribavirin, pentamidine)^†^	• (2 pairs)	•	•	• (If risk of splashing)		
Patient care	• (1 pair)	• (When at risk for exposure for bodily fluids)		• (If risk of splashing, e.g., disposal of bodily fluids)		
Management of extravasation	• (2 pairs)	•		• (If risk of splashing)		
Handling of contaminated bedding on the wards	• (2 pairs)	•				
Waste management (collection and transport)	(2 pairs)					
Spill or damaged or broken container	• (2 pairs)	•	• (If suspicion of powder or aerosolization is generated)	•		• (If on the floor)
Cleaning of sterile preparation room and airlock	• (2 pairs)	•			•	• (2 pairs)
Cleaning of preparation cabinets (hoods)	• (2 pairs)	•	•	•	•	•
Cleaning of other oncology pharmacy rooms and care units/clinics	• (1 pair)	•				

Abbreviations: G = gastric tube, J = jejunostomy tube, NG = nasal gastric tube, RPD= respiratory protection device.

*Although the risk of contamination with oral medications is minimal, the Working Group members believe that consistency of practice for any handling of hazardous drugs is of primary importance, and the preference is to wear a standard chemotherapy glove.

^†^ Although hazardous, they are not cytotoxic

#### Gloves

The gloves used to handle hazardous drugs are strongly recommended to comply with ASTM standard D-6978-(05)-13 and be powder-free.^11^ Gloves are recommended to be nitrile, polyurethane, neoprene or latex.^11^ Latex is a known allergen; therefore, it is strongly recommended that this be taken into consideration for glove selection. It is strongly recommended that vinyl gloves not be used.^12^ It is strongly recommended that the frequency of glove changes be adjusted according to the level of exposure at each step in the medication circuit. For example, when administering reconstituted medications, it is strongly recommended that workers change gloves immediately if torn, punctured or visibly contaminated with a hazardous drug, and follow Routine Practices.^13^ Gloves should be changed every 30 min unless otherwise recommended by the manufacturer's documentation.^3,4^ It is strongly recommended that great care be taken in the removal of gloves to not contaminate the skin. When two pairs of gloves are required, put on the first pair before putting on the gown.

#### Gown

It is strongly recommended that the gowns used for handling hazardous drugs be disposable, made of lint-free, low-permeability fabric, have long sleeves with tight-fitting cuffs and fasten in the back. Gowns need to be changed at the end of the procedure, in the event of contamination, spillage or rips. It is strongly recommended that the supplier be able to certify that the gown protects against hazardous drugs.^3,14^

For medication preparation and administration, gowns need to be changed halfway through a shift or every 3 and a half hours.^3,4^

It is strongly recommended that care be taken to avoid contamination of the hands by avoiding touching the outside of the gown when removing the gown.

#### Facial protection

Surgical/procedure masks are required while handling and preparing medications in a BSC and, in this instance, are worn to prevent microbial contamination of the sterile field.

Goggles and a face shield or full face-piece respirator should be worn when there is a risk of spills or splashes of hazardous drugs or hazardous waste materials when working outside of a BSC such as administration of hazardous drugs in the surgical suite, working at or above eye level or cleaning a spill.^3,4^

Head and hair coverings (including beard and moustache, if applicable) and sleeve covers provide protection from contact with hazardous drug residue. Disposable sleeve covers may be used to protect areas of the arm that may come in contact with hazardous materials. Disposable sleeve covers made of polyethylene-coated polypropylene or other laminate materials offer better protection than those made of uncoated materials.^4^

It is strongly recommended that full-facial protection be worn whenever there is a risk of splashing (e.g., during certain drug administration procedures). The use of a full-facial shield is preferred. If goggles are used, they need to be worn in conjunction with a fluid-resistant mask. For further information, see Canadian Standard Association (CSA) standard Z94.3-07 – Eye and Face Protectors.^15^ Eyeglasses alone or safety glasses with side shields do not protect the eyes adequately from splashes. Face shields in combination with goggles provide a full range of protection against splashes to the face and eyes. Face shields alone do not provide full eye and face protection.^4^

#### Respiratory protection devices

It is strongly recommended that fit-tested respirators such as NIOSH-certified N95 or N100 be used when there is a risk that airborne powder or aerosol will be generated. It is legislated that respirators be used in accordance with a respiratory protection program such as that outlined in CSA Standard Z94.4-18 “Selection, Use and Care of Respirators”.^16^

#### Caps

Caps are only required in the sterile preparation room and are worn to prevent microbial contamination of the sterile field.

#### Shoe covers

Disposable shoe covers are worn to prevent contamination of the healthcare workers’ shoes, and it is strongly recommended that they be worn when in the sterile preparation room or in the event of a spill. It is strongly recommended that shoe covers be removed immediately when leaving the sterile prep room to avoid contamination of other areas. When compounding hazardous drugs, the second pair of shoe covers must be donned before entering the Containment Secondary Engineering Control (C-SEC) and doffed when exiting the C-SEC.^3,4^

### Recommendation 3: receiving and transport

#### Handling hazardous drug delivery containers

It is strongly recommended that all receiving-dock workers receive training in the proper handling of hazardous drugs. It is strongly recommended that the receiving-dock workers check the integrity of the external packaging upon receipt; in the event of breakage or a damaged parcel likely to cause a spill, apply the Spill Protocol from your institution.

It is strongly recommended that delivery containers be taken immediately to the Pharmacy Department by the receiving-dock workers or the distributor.

It is strongly recommended that the receiving-dock or storeroom workers not open the delivery containers. It is strongly recommended that the delivery containers be handled with care to avoid breakage of the hazardous drug containers and not be left unattended in a corridor. Only trained workers (e.g., pharmacy technicians) are to proceed with the unpacking and subsequent steps.

#### Damaged containers/spill

It is strongly recommended that damaged containers be handled like spills. It is strongly recommended that the manufacturer or distributor be notified if the container is received in a damaged state. To limit exposure, it is strongly recommended that a damaged container not be returned to the manufacturer or distributor unless they require it returned. The damaged container will need to be returned in an impervious box. Notify the pharmacy if any damaged containers are suspected.^4^

See Recommendation 10: Management of Waste, Accidental Exposure, Spills and Returns.

#### Recommendation 4: unpacking and storage

Packaging can have high levels of contamination. It is strongly recommended that there be an unpacking area in the pharmacy limiting exposure risks. It is strongly recommended that the unpacking area be a separate dedicated space, separate from eating areas and preferably a separate room. It is regulated that there be adequate ventilation in the area, negative pressure and preferably vented to the outside. It is strongly recommended that there be a receptacle for hazardous waste in the unpacking area, for the disposal of secondary packaging.^3,6,17^

It is strongly recommended that workers at risk of exposure wear a protective gown and two (2) pairs of gloves when unpacking and cleaning hazardous drugs, from the opening of the external packaging to the placing of the secondary and/or primary packaging in their storage space. It is strongly recommended that workers check the integrity of all packaging at every step of the unpacking process. In the event of breakage or leaking, it is strongly recommended that the damaged contents be treated as a spill. It is strongly recommended that the primary and or secondary packaging be cleaned prior to being placed in storage.

It is strongly recommended that a regular cleaning protocol be in place either at this stage or prior to storage in the clean room. It is strongly recommended that all drug containers be cleaned to reduce external contamination. An example is the use of pre-moistened towelettes. It is important to ensure that the procedure does not damage the container or interfere with the reading of the label. It is also important to ensure that any product that is used will not further contaminate the product or work environment. However, it is strongly recommended that this procedure not increase the risk of incidents/accidents due to damage to the hazardous drug container or label.

It is strongly recommended that procedures be in place to minimize the risk of contamination of surfaces during the cleaning of vials (e.g., use of a disposable, plastic-backed, absorbent pad). It is strongly recommended that all surfaces be cleaned when the task is complete.

Establish a dedicated negative-pressure storage area for hazardous drugs that minimize the risk of contamination.^3^

When removing or transporting drugs out of the storage area, it is strongly recommended that one pair of gloves and a gown be worn and a spill kit be readily available.

#### Recommendation 5: planning the oncology pharmacy

It is strongly recommended that the oncology pharmacy be in compliance with relevant guidelines from the Canadian Society of Hospital Pharmacists and Accreditation Canada standards. Although the specific details of oncology pharmacy planning are beyond the scope of this document, details and some important considerations may be found in the National Association of Pharmacy Regulatory Authorities (NAPRA) guideline and CSA document CSA Z8000-11.^3,18,20^

It is strongly recommended that special requirements for heating, ventilation and air-conditioning systems in healthcare facilities be taken into consideration.^3,19^

A class II type B BSC is required with a preference for the type B2 because it ensures that there is no recirculation of air within the cabinet.^3,18,20^

There is emerging evidence suggesting some robotic devices that prepare hazardous drugs improve the accuracy of medication preparation and reduce potentially harmful staff safety events. Further studies are required to establish the cost-effectiveness of these robotic implementations. Each healthcare facility will need to assess the need for such devices in their environment.^20–24^

It is strongly recommended that all mixing and preparation of administration sets with a hazardous drug be performed in one centralized area in a specially designated class II type B BSC^21^ that:

- is exhausted through a HEPA filter to the outside atmosphere in a manner that prevents recirculation into any inside area;
- has exhaust and ventilation systems that remain in operation for a sufficient period of time to ensure that no contaminants escape from the BSC into the workplace; and
- is equipped with a continuous monitoring device to permit confirmation of adequate airflow and cabinet performance.

It is recommended that airlocks be considered if there are particular concerns about the propagation of airborne hazardous drugs.

It is strongly recommended that the priming of administration sets be prepared in the manner mentioned above.

It is strongly recommended that the layout allow and facilitate the unimpeded cleaning of all surfaces (walls, floors, ceilings, doors, diffusers, windows). It is strongly recommended that the furniture and equipment in the sterile preparation room be kept to a bare minimum. It is strongly recommended that there be a visual link; for example, a sealed window and a way to communicate between the sterile preparation room and the pharmacy, to view the work in progress. It is strongly recommended that access to the sterile room be limited to trained and authorized workers.^2^ A pass-through window can be installed to minimize the risk of contamination when transferring products into and out of the clean room. The pass-through should be equipped with an interlocking system or procedure that prevents both doors from being open at the same time.^3^

Limit worker traffic, particularly near unpacking and storage areas (to avoid accidental breakage) and near preparation cabinets (to avoid interfering with their proper operation).

It is legislated that the facilities include an emergency eyewash that may or may not be hooked up to the airlock sink.^10^ As a minimum, it is strongly recommended that an emergency eyewash be able to provide 15 min of flushing to both eyes.^23^ It is strongly recommended that a full shower be accessible nearby (e.g., in the oncology units/clinics).

Closed system drug-transfer devices are not a substitute for class II type B BSC. There is evidence from studies^24–48^ that closed-system drug-transfer devices can reduce contamination during preparation and increase or extend the beyond-use date of a drug. Further emerging evidence suggests that when these devices are not used as specified, they could become open to the environment. Further research is needed to evaluate this possibility.

In the non-sterile drug preparation process (e.g., oral preparations), it is strongly recommended that the same level of worker protection be adhered to.

#### Pharmacy policies and procedures

Establish policies and procedures regarding preventive maintenance, monitoring, certification and the optimal use of facilities and equipment.^49^

#### Recommendation 6: hazardous drug preparation

The following recommendations apply but are not limited to the preparation of all hazardous medications including parenteral, oral and topical, both sterile and non-sterile preparations. It is strongly recommended that policies and procedures include the use of appropriate PPE, the equipment for preparation including appropriate ventilation, and other automated equipment for packaging and a dedicated work area.

#### PPE

It is strongly recommended that workers (pharmacists or pharmacy technicians) wear a cap, surgical/procedure mask, shoe covers, a protective gown and two (2) pairs of gloves (see Table 1) to make sterile preparations of hazardous drugs in preparation cabinets.

#### Organization of the work

Organize the work to limit microbial and environmental contamination.

For both sterile and non-sterile preparations, it is strongly recommended that workers cover the work surface with a disposable, absorbent, sterile, plastic-backed pad to absorb any liquid contamination that may occur during handling. It is strongly recommended that the pad not cover the front and rear grilles of the preparation cabinet. It is strongly recommended that it be changed after 3.5 h of continuous work or for a new batch of preparations (e.g., a set of vials of a given drug) or in the event of a spill or contamination.^12^ It is legislated that the pad be disposed of in a hazardous waste receptacle.^4,8^

Limit the quantity of supplies and hazardous drugs in the cabinet, to avoid adversely affecting the laminar flow and to facilitate regular cleaning of the work surface. Place the sterile products in the centre and the non-sterile products (e.g., waste receptacle) along with the sides of the cabinet.

#### Removal of packaging

Remove the packaging, when applicable, and clean all the drug containers before taking them into the preparation cabinet. For sterile preparations, adhere to an aseptic technique for sterility.

#### Handling techniques

Use handling techniques that limit the risk of injury or accidental exposure. Direct CSTD spikes can be used to connect the hazardous medication bag directly to the tubing if spiking must occur at the bedside. When this adaptor is not used, IV bags containing hazardous drugs should only be spiked in a BSC to prevent exposure.

#### Preparation, priming and removing air from the tubing

It is strongly recommended that hazardous drugs be reconstituted in the pharmacy environment as described above. It is strongly recommended that the drug containers not be overfilled to avoid compromising the integrity of the container. It is strongly recommended that the techniques used for priming and removal of air minimize the exposure risks. It is recommended to only remove air from an IV tubing that does not contain a solution with a hazardous drug(s). It is strongly recommended that IV tubing is primed and air removed in the pharmacy, prior to adding the hazardous drug(s) to the infusion solution. Glass containers are not recommended due to the increased risk of breakage and exposure.

#### Labelling and final packaging

It is legislated that hazardous drugs be labelled to inform those handling these preparations of the nature of the drugs and the precautions to be taken. It is legislated that hazardous drugs display the “Cytotoxic” hazard symbol or the word “Cytotoxic”.^7,8^

It is strongly recommended that the outside surface of the hazardous drug containers (e.g., syringes, infusion bags, tubing) in the preparation cabinet be cleaned in the cabinet.

Place each hazardous drug container (e.g., syringe, bag), as well as the administration supplies (e.g., tubing), in a clear, leak-proof plastic bag to facilitate identification by the nurse without having to remove the container from the bag.

Following final verification in the pharmacy, it is strongly recommended that the plastic bags containing the hazardous drugs be placed in a rigid transport container (ideally opaque), properly identified with the “Cytotoxic” hazard symbol.^3,4^

#### Waste

It is strongly recommended that everything that comes out of the cabinet be wiped clean.

It is strongly recommended that all contaminated waste be disposed of in the chemotherapy waste stream.

### Recommendation 7: transport and storage following preparation

#### On-site transport of hazardous drugs

Transport hazardous drugs using a method that will prevent contamination of the environment in the event of breakage.

It is strongly recommended that hazardous drugs be placed in a closed, single-use leak-proof plastic bag.

It is strongly recommended that transport of the hazardous drug in a single-use closed, leak-proof plastic bag from the pharmacy to an area not adjacent to the preparation area (e.g., care unit, outpatient clinic), be done in a rigid, shock-resistant, leak-proof container made of a material that can be easily cleaned and decontaminated in the event of a drug leak.^20^ It is strongly recommended that the bottom be covered with an absorbent, plastic-backed cloth.

It is legislated that the transport container be identified with the “Cytotoxic” hazard symbol and be cleaned regularly.^7,8^ This container should be cleaned according to the protocol outlined by a committee responsible for hazardous drug handling.

It is strongly recommended that mechanical transport systems, such as pneumatic tubes, not be used because of the stress they put on the contents, and the whole transport system would be compromised if a leak occurred.^4,20^

It is strongly recommended that prepared medications be stored in a designated area prior to administration. It is strongly recommended that this area be cleaned regularly.

#### Off-site shipping and transport of hazardous drugs

Establish policies and procedures regarding the shipping of hazardous drugs.^50^

In the event that hazardous drugs are shipped off-site (e.g., from one institution to another), it is strongly recommended that they be packed separately from other drugs, according to the recommendations from the manufacturer and distributor. It is strongly recommended that pharmacy be consulted in the packaging of hazardous drugs.

It is strongly recommended that hazardous drugs be packed in a double plastic bag and placed in a box that is properly identified with the “Cytotoxic” hazard symbol. If necessary, immobilize the drug with packing^20,50^ material. It is legislated that the “Cytotoxic” hazard symbol be visible on the outside of the delivery^7^ container. It is strongly recommended that reusable delivery containers be cleaned regularly.

Ensure that the courier company will handle hazardous drugs.

#### Recommendation 8: drug administration

It is strongly recommended that safe handling and administration techniques be used to minimize possible exposure to individuals and the environment when administering hazardous drugs.

- It is legislated that appropriate PPE be made available to all healthcare workers and be worn as prescribed by the employer (Table 1).^10^
- It is strongly recommended that Luer lock connectors and needleless administration systems be used to administer any IV medications.
- Closed-system drug-transfer devices may offer additional protection.
- It is strongly recommended that disposable plastic-backed absorbent pads be used over work surfaces and placed under tubing or bag connections and ports when attaching any tubing, bag or syringe that has been exposed to a hazardous drug.
- Unless a closed system is used, never disconnect the tubing from hazardous drug bags. Discard bag with attached tubing into an appropriate waste container as a single unit.
- It is legislated that safety-engineered needles be used as per Needle Safety Regulation 474/07 made under the Occupation Health and Safety Act Labour, 2010.^51^ Do not purge air from the needle before administration.
- It is strongly recommended that oral hazardous drugs be handled in a manner that avoids skin contact, liberation of aerosols or powdered medicine into the air and cross-contamination with other^52^ medicines.
- It is strongly recommended that solid oral preparations (tablets) of hazardous drugs be crushed or cut within the BSC. If patients are unable to take in the solid format, it is strongly recommended that the pharmacy provide these drugs in an oral syringe or dissolve and dose container, in a ready-to-administer, liquid oral form.
- It is strongly recommended that the application of topical hazardous drugs be done using appropriate PPE and in a way that prevents contamination of the environment. Between applications, it is strongly recommended that the hazardous medication (i.e., tube or jar) be kept in a safe container and in a secure place that prevents contamination of the surrounding environment.
- With any intravesical administration, for example, bladder instillation, ensure there are detailed procedures in place to avoid risks of splashing.
- Use caution when administering intrathecal hazardous drugs, as there is a risk of splashing due to increased intrathecal pressures. A closed system (i.e., Luer lock) should be used when possible.

### Recommendation 9: home care

#### Home care of patients who have received hazardous drugs

It is strongly recommended that all hazardous drug preparations be compounded in pharmacies meeting the requirements for hazardous drug preparation.^20^

It is strongly recommended that hazardous drugs be transported, administered and disposed of by individuals who have received appropriate training. It is strongly recommended that hazardous drug transport containers are not reused by patients for domestic purposes, which may expose the family to cytotoxic drugs (e.g., toy box and sewing basket).

It is legislated that the healthcare provider who administers hazardous drugs in the home wear PPE as outlined in Table 1.^10^

It is strongly recommended that healthcare providers follow the same recommendations outlined in Recommendation 8 – Drug Administration

It is strongly recommended that a spill kit be readily available in the home in case of accidental spills.

It is strongly recommended that patients be informed of and be provided with written instructions and PPE for the safe handling of hazardous drugs.

It is strongly recommended that contact information be provided for home care patients who require assistance with the safe handling of hazardous drugs.

#### Hazardous drug waste in the home

It is strongly recommended that the institution have a clear process to address the issue of hazardous waste from patients in their homes, in compliance with municipal or local hazardous waste rules. It is strongly recommended that this process includes patient and caregiver education.

It is strongly recommended that caregiving staff provide the patients/caregivers involved in administering cytotoxic drugs in the home with a process for appropriate disposal of hazardous waste, including leftover drugs.

### Recommendation 10: management of waste

#### Bodily fluid waste

It is strongly recommended that workers who handle the biological fluids, excreta, contaminated bedding and soiled equipment of patients who have received hazardous drugs wear two (2) pairs of gloves and a protective gown. It is strongly recommended that face protection be worn when there is a risk of splashing.

#### Cytotoxic drug waste

Establish policies and procedures as per provincial legislation regarding hazardous waste management.

The term “hazardous waste” includes any material that comes into contact with hazardous drugs during their storage, handling, preparation, administration and disposal (e.g., packaging material, protective equipment, preparation supplies, such as syringes, tubing, drug bags; soiled disposable incontinent briefs of patients who have received hazardous drugs during the previous 48 h or longer depending on the drug; and hood pre-filters and HEPA filters).

It is legislated that hazardous waste is placed in a waste container clearly identified with the “Cytotoxic” hazard symbol. It is legislated that hazardous waste be disposed of in the appropriate containers.^8^

It is legislated that sharps be placed in rigid containers with a leak-proof lid; CSA standard Z316.6–07 specifies the use of the colour red for the rigid containers.^53^ If the containers are another colour, follow the instructions of the company ensuring the final disposal.^8^

It is strongly recommended that other wastes (soft items, such as tubing and protective equipment) be placed in leak-proof and tear-resistant containers, identified with the “Cytotoxic” hazard symbol.^4^

For final disposal outside the institution, it is legislated that all hazardous waste is in a rigid, leak-proof, container identified with the “Cytotoxic” hazard symbol and scheduled for transport outside the institution.^8^

It is legislated that any excess fluid from hazardous drugs (e.g., drug loss) be disposed of in a sealed container and placed in a rigid container, the bottom of which is to be covered with an absorbent pad. This rigid container will be handled like other hazardous wastes.^8^

It is recommended that disposable/incontinent briefs soiled by patients who have received hazardous drugs be placed in a hazardous waste container.

It is legislated that hazardous waste be incinerated according to ministry guidelines.^8,54^

It is legislated that hazardous waste not be disposed of in the receptacles used for infectious biomedical waste (which may be autoclaved and then sent to a landfill site).^8^

It is legislated that every area where hazardous drugs are handled will have an appropriate hazardous waste receptacle as close as possible to the work area.^8^

The lids of hazardous drug receptacles must remain closed, except when depositing waste. Bins with foot pedals and lids, which lock automatically when full, are recommended to minimize exposure.

It is strongly recommended that workers be careful to avoid contaminating the outside of the receptacle when depositing waste.

It is legislated that the transport of hazardous waste receptacles be assigned to properly trained workers.^6^

It is strongly recommended that workers who handle hazardous waste receptacles wear two pairs of disposable gloves and have a spill kit at their disposal. It is strongly recommended that the waste go through as few care units, public areas and areas containing food or linens as possible.

It is legislated that the final storage areas for hazardous waste receptacles be secure. Refer to Ontario storage^7,8^ requirements.

#### Recommendation 11: accidental exposure

Be aware of any mandatory reporting requirements under the Occupational Health and Safety Act and report requirements to Workplace Safety and Insurance Board (WSIB).^6^

Establish policies and procedures regarding accidental worker exposure.

If a hazardous drug accidentally comes into contact with a worker's skin or clothing, it is strongly recommended that the worker immediately remove the contaminated clothing and thoroughly wash the skin of the affected area with soap and water and continue to rinse for 15 min. If appropriate, it is strongly recommended that the contaminated worker take a shower. It is strongly recommended that a deluge shower be made available in the vicinity (e.g., in the oncology clinics/units). It is strongly recommended that all contaminated clothing be discarded in hazardous waste. Workers should seek medical attention after exposure.

If a hazardous drug comes into contact with a worker's eyes, it is strongly recommended that the worker flush their eyes at an eye wash station. Alternatively, it is recommended that the workers use an isotonic solution to flush their eyes (e.g., sterile NaCl 0.9%). It is strongly recommended that eyes be flushed for at least 15 min.^19^ It is strongly recommended that if contact lenses are worn, they be removed immediately prior to flushing. Workers should seek medical attention after eye exposure.

In the event of a needlestick or sharps injury, let the wound bleed freely. Under running water, gently and thoroughly wash the area with soap. Contact Occupational Health. Ensure that facility policies for needlestick or sharps injury are followed including completion of an incident report and reporting to WSIB if indicated.

#### Recommendation 12: spills management

It is strongly recommended that the facility develops policies and procedures for spills management that takes into account the types of spills (i.e., amount, location, concentration and powder vs. liquid), incidence reporting, surveillance of spills and restocking of equipment.

All staff working in environments where hazardous drugs are handled should be trained in the use of a spill kit.

It is strongly recommended that a spill kit be readily available in all areas where hazardous drugs are stored, transported, handled and administered.

It is strongly recommended that a spill kit be readily available in the home in case of accidental spills, but institutions must ensure patients, or their caregivers are trained on the use of the spill kit and PPE.

It is legislated that disposable items from the clean-up of spills be placed in the hazardous waste receptacle.^8^ Non-disposable items should be thoroughly cleaned and decontaminated.

The area of the spill should be decontaminated deactivated and disinfected.^3^ According to NAPRA: “Decontamination involves the transfer of a hazardous drug contaminant from a fixed surface (e.g., counter, bag of solution) to a disposable surface (e.g., wipe, cloth). The wipe is then contained and discarded as hazardous waste. Many solutions can be used for decontamination, for example, 70% isopropyl alcohol, sterile water, hydrogen peroxide and sodium hypochlorite.”^3^ “Deactivation is the treatment of a hazardous drug to create a less hazardous agent, for example, by chemical deactivation. The material safety data sheets for some hazardous drugs recommend sodium hypochlorite for this purpose, usually as a 2% solution. This compound will corrode stainless steel surfaces, so it must then be neutralized with sodium thiosulphate or removed with a germicidal detergent. Sodium hypochlorite also has an additional germicidal effect for disinfection.”^3^ “Disinfection is the process of destroying microorganisms.”^3^

Most spills can be contained and managed by trained staff (e.g., leaking IV tubing).

When a spill is not contained or easily managed (e.g., exposure to large volume of fluid that is a risk to the environment or a large crate of vials filled with powder broken in the receiving area), it is strongly recommended that a Code Brown or equivalent be called.

#### Recommendation 13: environmental cleaning

Establish environmental cleaning policies and procedures for all surfaces where contact with hazardous drugs may occur. Areas should be decontaminated, deactivated and disinfected following legislative procedures Examples may include unpacking and storage, preparation, administration and disposal areas. Pharmacy counters are among the most contaminated surfaces.^3,4,20^

It is strongly recommended that cleaning of the BSC be performed by trained personnel following manufacturer's and NAPRA's guidelines.^3,4^

#### Use of pumps to administer hazardous drugs

Make sure there is an appropriate policy to clean and inspect the equipment between uses.

#### Laundry

Ensure the facility complies with the Occupational Health and Safety Act – Ontario Regulation for Health Care and Residential Facilities.^6^ Contaminated items should be placed in sealable bags and washed separately from other items.^20^

### Recommendation 14: medical surveillance and environmental monitoring

#### Medical surveillance

Methods used to investigate the potential health effects of exposure to hazardous drugs are inconclusive and difficult to interpret. The ideal test should meet several requirements – it should be sensitive, specific, quantitative, rapid and reproducible. Importantly, the procedures for taking a sample should be non-invasive and should not cause unnecessary duress or anxiety to the individual.^4^

Unfortunately, there is currently no suitable test to meet these requirements. Therefore, there is conflicting information and opinion about the value of routine biological monitoring for employees handling hazardous drugs.

Employers do have a responsibility to ensure that they remain aware of and apply any future developments for monitoring the health of employees in the handling of hazardous drugs.

The panel supports further research to determine whether there are adverse health effects that result from exposure to hazardous drugs.

Adherence to agreed standard operating procedures with sufficient initial and regular ongoing training in safe handling/administration is paramount to reducing the potential for exposure and risk.

There is evidence in the literature of a higher rate of spontaneous abortion among women working in roles that expose them to hazardous drugs.^55–60^ There are no other identified medical conditions known to result from chronic exposure of healthcare workers to hazardous drugs, no exposure limits set for hazardous drugs and no standards for the interpretation of test results of exposed healthcare workers to enable meaningful interpretation or action based on biological monitoring results.

#### Environmental monitoring

It is recommended that the facility implement an environmental monitoring program. Surface testing would audit contamination of the environment (e.g., pharmacy counters, patient bedside tables) and provide a quality indicator of cleaning effectiveness and adherence to recommended work practices.^20^
